# Intra-adaptational changes in online adaptive radiotherapy: from the ideal to the real dose

**DOI:** 10.1007/s00066-025-02425-9

**Published:** 2025-07-15

**Authors:** Hanna Malygina, Hendrik Auerbach, Marc Ries, Frank Nuesken, Bryan Salazar Zuniga, Sobhan Moumeniahangar, Florian Oeschger, Markus Hecht, Jan Palm, Yvonne Dzierma

**Affiliations:** 1 Department of Radiotherapy and Radiation Oncology, Saarland University Medical Centre, Kirrberger Str. 100, 66421 Homburg/Saar, Germany; 2 Department of Radiotherapy, Rostock University Medical Center, Rostock, Germany; 3 Clinic for Radiation Oncology, Canton of St. Gallen-Hospital, St. Gallen, Switzerland

**Keywords:** Prostate cancer, oART, Varian Ethos, Intrafractional changes, Dosimetric impact

## Abstract

**Background and purpose:**

Online adaptive radiotherapy has demonstrated dosimetric benefits by accounting for interfractional organ variations. However, this study investigates the dosimetric impact of intra-adaptational anatomical changes that take place during the adaptation process.

**Methods:**

Our retrospective analysis was conducted on 155 fractions from 8 prostate cancer patients treated with adaptive radiotherapy using the Varian Ethos system (Varian, Palo Alto, California, USA). Various dose–volume metrics for the targets and organs at risk were assessed for (1) the non-adapted (an original plan on a pretreatment cone-beam CT [CBCT], acquired at the beginning of a treatment session), (2) the adapted (an adapted plan on a pretreatment CBCT), and (3) the delivered dose distributions (an adapted plan on a pre-irradiation CBCT acquired for patient position verification with recontoured organs).

**Results:**

For the target metrics, we quantitatively proved that the delivered dose distribution was still beneficial in comparison to the non-adapted one, despite the anatomical changes during the adaptation process. The bladder dose–volume metrics strongly depended on the bladder volume variations across the planning CT and both CBCTs, frequently showing improvement during the adaptation process as the bladder continued to fill. In contrast, no clear trend was observed for the rectum or posterior rectum wall metrics. In only a small fraction of sessions (up to 5% for most metrics) were the metric objectives not achieved with the delivered dose while they were achieved with the adapted one. Physiological reasons for these occurrences stemmed from meteorism occurring between pretreatment and pre-irradiation CBCTs.

**Conclusion:**

This study confirms that the dosimetric advantages of online adaptive radiotherapy persist in clinical practice, despite anatomical changes due to the time delay needed for the adaptation process.

**Supplementary Information:**

The online version of this article (10.1007/s00066-025-02425-9) contains supplementary material, which is available to authorized users.

## Introduction

**Fig. 1:**
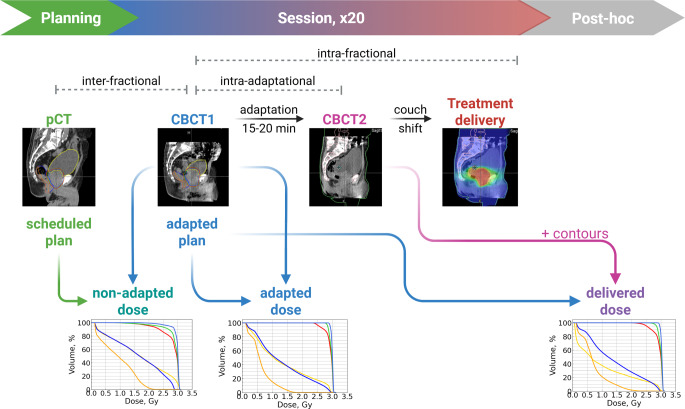
Flowchart depicting the online ART workflow (planning and session, x20) and the workflow for this study (post-hoc). The latter includes contouring on CBCT2 (cone-beam CT) and calculation of DVH (dose-volume histogram) for the delivered dose. pCT refers to planning CT. Created in BioRender. Malygina, H. (2025). https://BioRender.com/z97b035

**Fig. 2:**
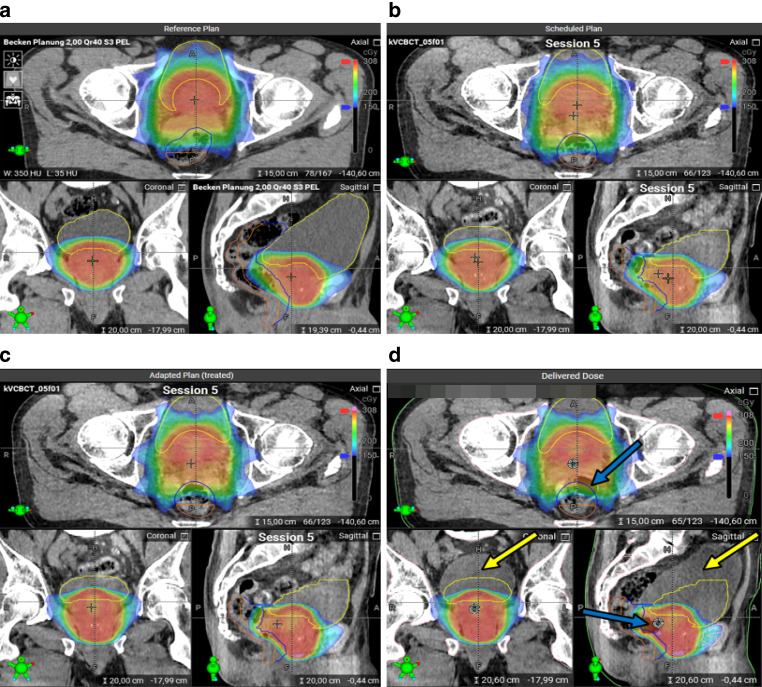
Original and adapted plans on different (CB)CT images for the same treatment session. **a** The planning CT with the original treatment plan. **b** The original plan on CBCT1. **c** The adapted plan on CBCT1. **d** The adapted plan on CBCT2, with contours taken from CBCT1. As can be seen, particularly the bladder has changed its volume between CBCT1 and CBCT2, and an air pocket has moved down the rectum. Parts of both OARs lie outside of the contours (the yellow arrows point to the bladder part that is outside of the bladder contour, and the blue arrow to the rectum part). Patient 4, session 5. Contour color scheme: bladder—yellow, rectum—blue, posterior rectum wall—orange

To be able to apply a daily optimized dose distribution to the real patient anatomy was a long-cherished dream in radiotherapy, which has finally come true in recent years with the introduction of online adaptive radiotherapy (ART). The kV cone-beam CT (CBCT)-based ART workflow provided by the Varian Ethos system (Varian, Palo Alto, California, USA) [1, 2] is organized as follows: just as in traditional percutaneous radiotherapy, a planning CT (pCT) is acquired, on which the target and organs at risk are contoured and a dose prescription is defined (Fig. 1). This “treatment intent” is then realized by creating a template treatment plan by choosing from predefined scenarios (intensity-modulated radiotherapy with different equidistant beam arrangements or volumetric modulated arc therapy), which is accepted for treatment (Fig. 2a). Most likely, this accepted plan will never be delivered, since the daily anatomy of the patient will most probably never be identical to the planning CT.

At each treatment session, the patient is positioned for treatment and then a kV-CBCT is acquired to have the daily anatomy depicted (henceforth called CBCT1). After checking the image quality and registration, the system will propagate the contours to the new anatomy (where they must be revised and can be changed by the user) and provide the user with two dose distributions for comparison: the dose distribution that would result if the original plan were delivered to the actual anatomy (“scheduled plan on CBCT1,” sch@cbct1, “non-adapted dose”; Figs. 1 and 2b) and the automatically reoptimized adapted treatment plan that the system creates with the aim of reproducing the original treatment intents on the changed anatomy (“adapted plan on CBCT1,” adp@cbct1, “adapted dose”; Figs. 1 and 2c). Both dose distributions are hence calculated on the basis of CBCT1, representing the real anatomy at the moment the patient is imaged at the beginning of the fraction when the workflow begins. The user can then compare the dose distributions and dose–volume histogram (DVH)/metrics to decide which plan is better for the present-day anatomy. A number of studies [3–6] have shown that the adapted plan can balance out imperfections that arise from applying the predefined plan to a changing anatomy, such that in most cases, the “adapted plan on CBCT1” will outperform the “scheduled plan on CBCT1.” The treating physician will now choose the better-suited plan and accept it for treatment. For the sake of simplicity, in this work, we assume that the adapted plan will always be chosen, since it will be at least as good as the non-adapted plan. In fact, this corresponds to our in-house experience: out of 950 treated fractions so far, the adapted plan was chosen for treatment in 947 fractions and the scheduled plan in 3 fractions (0.3%).

Before proceeding to treatment, a final kV-CBCT (CBCT2) can optionally be acquired to ascertain that the patient’s position has not changed. However, no further adaptation can be performed on CBCT2. Now there is the rub: since the adaptation is a time-consuming process, some changes in anatomy can occur between CBCT1 and CBCT2 [7–9], and the real dose delivered to the patient will be that of the chosen plan on CBCT2 rather than on CBCT1 (“adapted plan on CBCT2,” adp@cbct2, “delivered dose”; Fig. 1 “Post-hoc” and Fig. 2d). The clinical workflow at our institution does not include a posttreatment CBCT; therefore, we deem CBCT2 to be the closest to the anatomy during plan delivery (the latter itself lasts about 3 min). Quite possibly, the dose distribution on CBCT2 might not be quite as good as the one optimized for CBCT1. Unfortunately, the effect of the change between CBCT1 and CBCT2 is not directly visible to the user. Although the final dose for evaluation and also dose accumulation is actually calculated on CBCT2, the contours are only rigidly propagated to this CBCT. Essentially, the contours from CBCT1 are used by the Ethos treatment planning and evaluation module to assess the DVH metrics of the delivered dose distribution. However, these metrics may not accurately reflect the real DVH values if the organ’s shape or volume has changed between CBCT1 and CBCT2.

The aim of this work is to compare the real delivered dose from the adapted plan (adapted plan on CBCT2, adp@cbct2) with the intended (ideal) adapted dose (adapted plan on CBCT1, adp@cbct1). How much accuracy is lost due to the anatomical change? While possibly not quite as good as the intended adapted plan, will the delivered dose still be better than in a non-adaptive workflow (i.e., will the adapted plan on CBCT2 still be better than the scheduled plan on CBCT1)? To stress this point, as CBCT2 is not required in the clinical workflow, many institutions may omit this second CBCT and might not be aware that the delivered dose is not identical to the intended dose. We believe that this issue is of paramount importance when assessing the benefit of adaptive radiotherapy, and, to our knowledge, only very little information is available on the subject.

## Patients and methods

### Treatment characteristics

Between July 2023 and March 2024, a total of 950 sessions/39 patients were treated with the Varian Ethos system at our institution. The majority of patients underwent pelvic radiotherapy, primarily for prostate cancer. Patients with primary prostate cancer radiotherapy are treated in our institution with the in-house protocol based on the CHHiP trial [10].

For this post hoc analysis, we selected all 18 patients with a moderate or high risk of seminal vesicle involvement, as defined in the CHHiP trial [10]. These patients had been treated prior to the commencement of this study, making this an exploratory analysis.

Treatment details, such as target volume definitions (Planning Target Volume [PTV] as well as both Simultaneous Integrated Boosts [SIB1, SIB2]) and prescribed doses, have been described previously [11] (see also Table 1). PTV, SIB1, and SIB2 are structures derived from prostate and seminal vesicles contours, which are necessary for the adaptive treatment workflow since they are automatically generated by the system based on the anatomically distinguishable adapted prostate and seminal vesicle contours.

**Table 1:** In-house clinically relevant dose constraints for the organs at risk and the target volumes considered in this study

Structure	Metric	Constraint	
		Optimal	Alternative
PTV	V95%	$\geqslant100\%$	$\geqslant95\%$
	D95%	$\geqslant98\%$	$\geqslant95\%$
SIB1	V95%	$\geqslant100\%$	$\geqslant95\%$
	D95%	$\geqslant98\%$	$\geqslant95\%$
SIB2	V95%	$\geqslant100\%$	$\geqslant95\%$
	D95%	$\geqslant98\%$	$\geqslant95\%$
Rectum	V48Gy	$<27\%$	$<35\%$
	V24Gy	$<70\%$	–
Bladder	V60Gy	$<5\%$	–
	V48Gy	$<25\%$	–
	V40Gy	$<50\%$	–
Posterior rectal wall	V37Gy	$<5\%$	–
	V30Gy^a^	$<5\%$	–

Both optimal and alternative (if available) constraints are provided for each metric

VxGy refers to the relative volume of the organ receiving x Gy or more, while Dx% refers to the relative dose that covers x% of the organ volume

The prescribed doses are defined individually for each PTV (planning target volume) and SIB (simultaneous integrated boost)

^a^PRW (posterior rectal wall) V30Gy is not clinically relevant and serves only for plan optimization

Dose objectives for organs at risk (OARs) in this study were aligned with our institution’s in-house protocol, which is based on the guidelines from the CHHiP [10], PROFIT [12], PACE‑B [13], and PACE‑C [14] trials. In our institution, the posterior rectum wall (PRW) is used as an additional OAR in order to obtain better dose shaping and to avoid a high dose for the whole rectum circumference, since a high dose to the rectum wall is associated with late rectal toxicities [15–17]. An automated algorithm derives the PRW contour from the rectum contour^1^. The specific OAR constraints used in this analysis are detailed in Table 1. All selected metrics are clinically relevant except for V30Gy for the PRW. This additional metric was included for better observation of dose variations in the PRW, as the clinically relevant Dmax and V37Gy typically exhibit very small values, often approaching zero. Furthermore, Dmax is highly sensitive to contouring quality.

To ensure better bladder sparing, patients are instructed to follow our in-house bladder and bowel preparation instructions, which include the following steps: (1) naturally void the bladder and the bowel; (2) drink 350 ml of liquid within 10 min; (3) engage in light physical activity for 1 h (e.g., walking); (4) attend the appointment (either a planning CT or a CBCT).

### Patient selection

The primary exclusion criterion pertains to prostate contouring. It is an inherent bias that the prostate contour volume on the pCT tends to be significantly smaller than on CBCT1 and CBCT2. This should not be regarded as a mistake or inconsistency, but rather as arising from the ESTRO ACROP contouring guidelines [18], which recommend assuming that the levator ani muscles next to the prostate will have the same thickness as next to the rectum when contouring on CT, while they can be distinguished from the prostate when contouring on MRI. Hence, the authors conclude that this “unequivocally leads to smaller target volumes, as unnecessary inclusion of the levator ani muscles is avoided” when contouring with the aid of MRI rather than CT only. Similarly, in CT-only-based contouring, Santorini’s plexus will often be included in the prostate contour when it cannot be clearly distinguished, again resulting in larger prostate and hence CTV and PTV volumes when no MRI is available. In our institution, the MRI images are used for creating the contours on the planning CT but are not always considered within the adaptive workflow, resulting sometimes in larger prostate contours for the adapted contours. The size of this effect is somewhat dependent on the patient. For large discrepancies, we expect this to potentially lead to several artifacts:

- The scheduled plan on CBCT1 (sch@cbct1), which evaluates the original dose distribution on the basis of the adapted contours, will appear to provide poorer target coverage even if no anatomic changes have occurred, simply because of the apparently larger CTV and PTV, which will not be perfectly covered by the prescribed isodose.
- On the other hand, the scheduled plan (whether on the pCT or on CBCT 1) will appear to provide better bladder and rectum sparing (particularly considering the high-dose metrics) even in the absence of any anatomical changes, because the smaller target volume will lead to less dose spill into the adjacent organs.

**Fig. 3:**
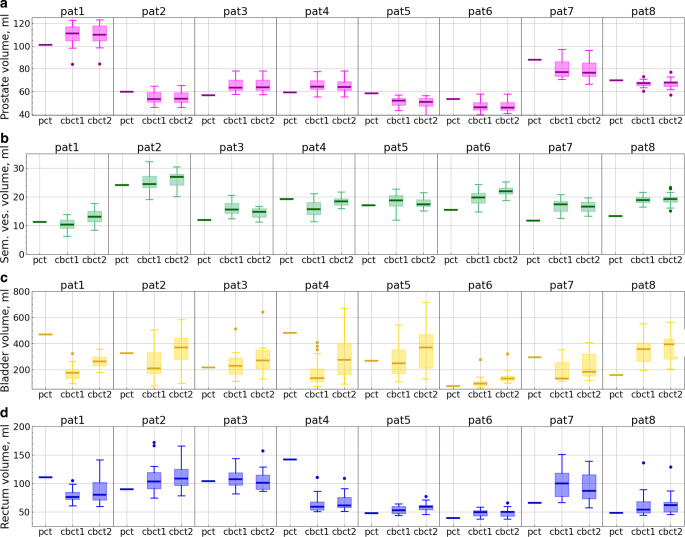
Organ volume distributions for each patient on pCT, CBCT1, and CBCT2 displayed across four panels: prostate (**a**), seminal vesicles (**b**), bladder (**c**), and rectum (**d**). The x‑axes represent the image used for contouring: “pct” corresponds to a planning CT (one volume value per patient), while “cbct1” and “cbct2” correspond to CBCT1 and CBCT2, respectively. The latter two contain up to 20 volume values for each patient, depending on the number of included sessions. Each box extends from the first quartile (Q1) to the third quartile (Q3), with a line indicating the median. Dots represent outliers (data points lying outside the interval $[Q1-1.5IQR, Q3+1.5IQR]$, where IQR denotes the interquartile range)

For this reason, the following exclusion criterion was applied: patients with a large discrepancy in prostate contour volumes between the CBCTs ($V_{i,\mathrm{CBCT}} | i = 1,2,..,N$; where *N* is the number of included sessions for a patient) and the pCT ($V_{\mathrm{pCT}}$) were excluded from the analysis to minimize the impact of this variability (for the prostate volumes, see Fig. 3). A threshold of 20% was arbitrarily selected for the following variable: $\sigma_{\mathrm{prost}} = {\frac{1}{V_{\mathrm{pCT}}}\sqrt{\frac{1}{N}\sum_{i=1}^{N}(V_{i,\mathrm{CBCT}} - V_{\mathrm{pCT}})^2}}$. With this criterion, 8 patients were excluded, with $\sigma_\mathrm{prost}$ ranging from 24 to 75% (some of them also had poor CBCT quality). Notably, all these patients had significantly smaller (not bigger) prostate contours on the pCT compared to the CBCTs.

Other exclusion criteria were applied to both patients and individual treatment sessions. Two patients who consistently exhibited poor CBCT quality were excluded from the analysis (one patient consistently had a large amount of bowel gas, which made precise contouring difficult; in the second patient, image artifacts—presumably due to motion during CBCT acquisition—compromised image quality). Additionally, single sessions with poor CBCT quality, interrupted sessions, and sessions where the scheduled plan was selected for treatment, were excluded, leading to the exclusion of 5/160 sessions in total (3%).

The semi-automated algorithm for PRW contouring mentioned above was introduced first in October 2023. Thus, for the PRW analysis, we included only those patients for whom this algorithm was implemented, to provide more self-consistency (7/8 patients; patient 5 is excluded).

In total, 8 patients and 155 sessions were analyzed (for the PRW analysis, 7 patients and 135 sessions).

### Data analysis

#### Data preparation

Since the study was retrospective, the data (contours and DVHs) for adp@cbct1 and sch@cbct1 were available before the study began. We needed to contour the organs on CBCT2 and calculate the DVHs with the adapted plan (adp@cbct2). Prostate and seminal vesicle contours were copied from CBCT1 to CBCT2 and manually corrected, keeping the changes as small as possible, as we did not expect them to be drastic. The OARs were contoured by the predefined auto-contouring workflow “Pelvis” in MIM (Version 7.4.2, MIM Software Inc., Cleveland, OH, USA) [19] and corrected by a user. To obtain self-consistent results, DVHs for all three dose distributions were calculated in MIM.

#### Statistical analysis

To address the research questions posed in this study, we analyzed the dose–volume metric distributions (listed in Table 1) for each plan–CBCT combination: sch@cbct1, adp@cbct1, and adp@cbct2, which correspond to the non-adapted, adapted, and delivered dose, respectively. We compared the distributions for adp@cbct1 versus adp@cbct2 as well as for adp@cbct2 versus sch@cbct1.

For each comparison, we applied the Wilcoxon signed-rank test for paired data with a two-sided alternative hypothesis on an individual patient basis to obtain the significance of the difference between the considered plan–CBCT combinations. Each patient had a maximum of 20 sessions included in the study (thus, 20 or fewer paired data points, *N*); therefore, reliable *p*-values were obtained either by employing the “exact” method of *p*-value calculation (possible only if a distribution does not contain zeros and all elements are unique) or by performing a permutation test with 10,000 permutations. Zeros were treated with the “zsplit” method from the Python SciPy library (see [20] for more details).

We calculated the medians of the distributions of metric differences between two doses (for example, for adp@cbct1 and adp@cbct2: $$\mathrm{Median}\,(\lbrace \mathrm{metric}_{\mathrm{adp@cbct1},i}\\ -  \mathrm{metric}_{\mathrm{adp@cbct2},i}\,|\,i=1,2,...,N \rbrace)$$) to assess which dose generally resulted in larger values for a specific metric.

The Wilcoxon signed-rank test operates on rank differences between paired data, meaning that absolute values are not considered. This enabled us to extend the test to the entire cohort, which increased the number of data pairs to a maximum of 155 (135 for the PRW analysis), thereby improving the *p*-value reliability.

A custom Python script was developed for this analysis, utilizing essential libraries such as NumPy, SciPy, statistics. Due to the exploratory nature of this study, *p*-values are considered descriptive, with a $p\!<\!0.05$ regarded as indicative of statistical significance.

## Results

### Intra-adaptational anatomical changes

#### Prostate

The prostate contour volume remained largely unchanged during the short interval between CBCT1 and CBCT2 (approximately 15–20 min, see Fig. 1 in the Supplement), with the mean volume difference between the two scans per patient ranging from 0.01 to 0.73 ml (Fig. 3).

#### Seminal vesicles

Similarly, no significant changes in the seminal vesicle volumes are anticipated between CBCT1 and CBCT2; the observed mean differences ranged from 0.12 to 2.8 ml across individual patients (Fig. 3).

#### Organs at risk

Changes in bladder and rectum volumes have physiological reasons, additionally to variations in contouring. For the bladder, we saw a systematic increase in volume between CBCT1 and CBCT2 (Fig. 3), which is plausible since bladder filling continued during the treatment session. The mean difference in bladder volume between CBCT2 and CBCT1 across patients ranged from 19.5 to 130.9 ml (the increase was statistically significant for each patient, with $p<0.001$ according to the Wilcoxon test), and the mean filling rate ranged from 1.14 to 5.92 ml/min.

For the rectum, no such systematic effect could be discerned (Fig. 3), with mean volume differences between CBCT1 and CBCT2 staying rather small and nonsignificant for most patients: between −3.6 and 6.3 ml, with $p > 0.1$ (except for patient 7 with an always full rectum, who exhibited a significant decrease in rectum volume, with mean values of 8.9 ml and $p=0.003$). However, in addition to some variability in manual contouring, we observed changes in shape due to peristalsis and movement of air pockets (see below).

### Dosimetric impact

#### Examples of two patients

**Fig. 4:**
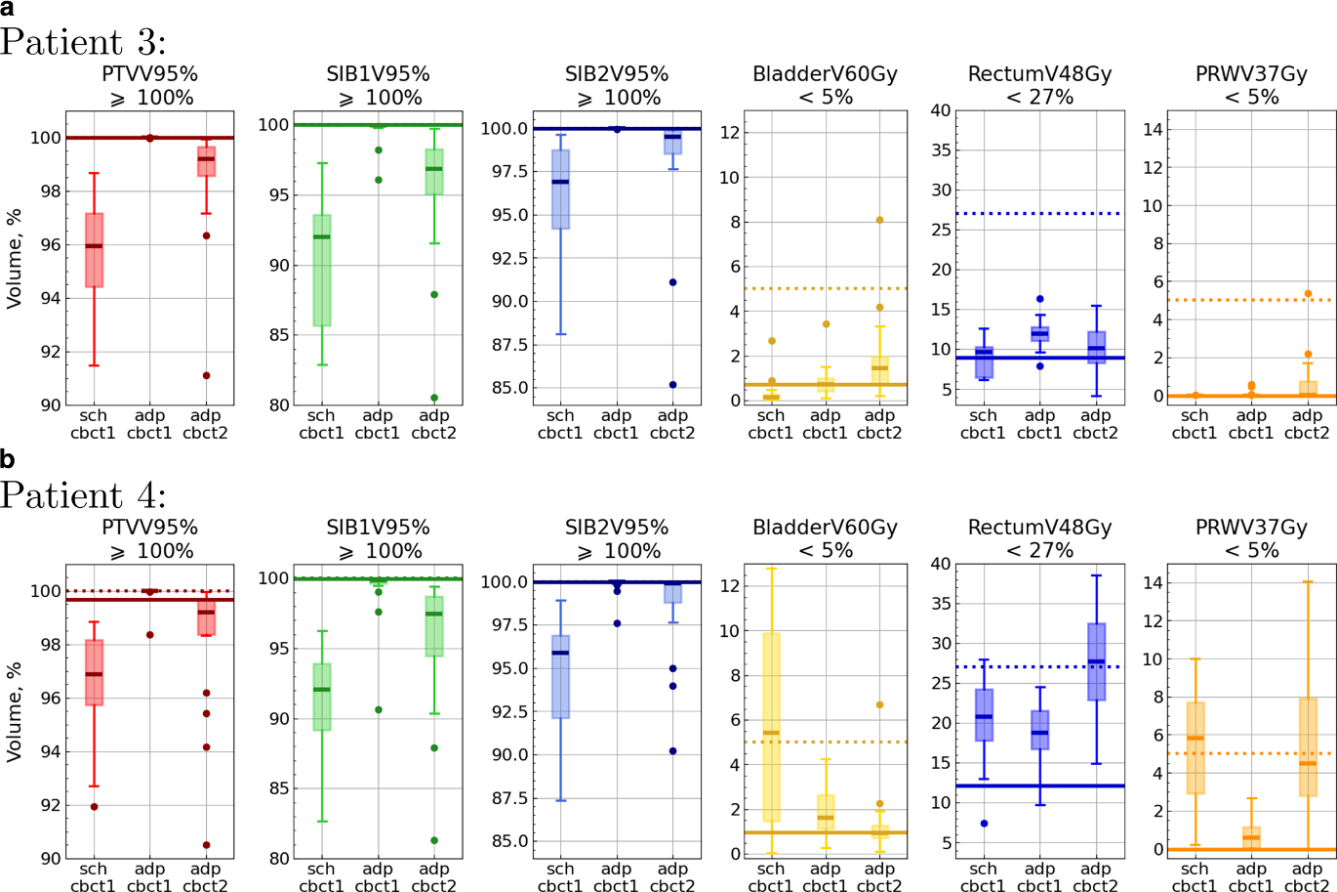
Metric distributions for patient 3 (**a**) and for patient 4 (**b**). The y‑axes present the metric values, while the x‑axes show the combination of a plan (“sch” or “adp”) and a CBCT (“cbct1” or “cbct2”). Bold lines represent the metric value with the scheduled plan on the pCT. Dotted lines correspond to the objective for each metric. Each box extends from the first quartile (Q1) to the third quartile (Q3), with a line indicating the median. Dots represent outliers (data points lying outside the interval $[Q1-1.5IQR, Q3+1.5IQR]$, where IQR denotes the interquartile range). The metric name and its objective are provided on top of each plot

Examples of the metric distributions for single patients (3 and 4) are depicted in Fig. 4. We comment in some detail on these plots, as they provide a representative and intuitive view of the most relevant effects.

For both these patients, it is evident that the scheduled plan will result in poorer dose coverage when evaluated on CBCT1 (which is, in part, due to the contouring method for the prostate, as described above; see also Fig. 3 for the prostate volumes). Regardless of the origin of this discrepancy, it will be considered a “real underdosage” in the adaptive workflow, and the aim of the adaptation will be to correct this. Consequently, the reoptimized, adapted plan (adp@cbct1) will again provide perfect target coverage, as defined in our treatment intents, i.e., 100% of the PTV/SIB1/SIB2 volume covered by 95% of the prescribed dose to this volume. In a way, this would correspond to a scaling of the plans in a planning study, and the adapted plan (on CBCT1) will always strive for this target coverage. In comparison with the ideal adapted plan (on CBCT1), the delivered dose (adp@cbct2) falls well short of this coverage, while remaining better than the scheduled plan. This effect is quite strong, as the loss in coverage between adp@cbct1 and adp@cbct2 *cannot* be explained by a change in prostate and target volume contouring. Since both adp@cbct1 and adp@cbct2 were contoured based on CBCT images only, these target volumes should be consistent with one another and no marked deviation in dose should ensue.

Regarding the OAR doses, the dose–volume metrics reflect the general changes in organ volumes as outlined above. However, these two patients represented different cases. Patient 3 had non-ideal bladder filling on the planning CT (Fig. 3). The bladder showed a small change between CBCT1 and CBCT2; thus, the metrics could not improve due to geometrical effects. Although all the bladder metrics stayed below the limits, significant degradation was observed with adp@cbct2 in comparison to adp@cbct1. Somewhat better performance of the scheduled plan on CBCT1 might be caused by a smaller prostate on the pCT: the scheduled plan had a smaller high-dose region (an example of such a session is shown in Fig. 2 in the Supplement) that expanded into the bladder (and the rectum).

For the rectum, no relevant change in volume or in dose metrics can be seen between CBCT1 and CBCT2 for patient 3 (although the differences for the rectum metrics were statistically significant, all the metrics remained well under the limits). There was slightly more dose to the PRW on CBCT2 because the plan was not optimized on the CBCT2 anatomy but rather on the CBCT1 anatomy.

In contrast to patient 3, patient 4 had high bladder filling on the planning CT (Fig. 3), but rather low filling on the CBCTs. This explains the very poor performance of the scheduled plan applied to the CBCT1 contours. Since the bladder volume on CBCT2 is larger than on CBCT1, the dose metrics will appear improved on CBCT2 due to geometry, even with a “less-adapted” plan.

Also for patient 4, no relevant change in rectum volume was observed between CBCT1 and CBCT2. The dose metrics showed, however, a notable deterioration with the adapted plan on CBCT2. Partially, this can be attributed to the fact that the plan was not optimized on the CBCT2 anatomy. Additionally, this very patient exhibited substantial changes in rectum shape between CBCT1 and CBCT2 (one session is depicted in Fig. 2), which impacted both the rectum and the PRW metrics.

#### Adapted vs. delivered dose distributions

This comparison addresses the question of how the anatomical changes between CBCT1 and CBCT2 influence plan accuracy.

**Fig. 5:**
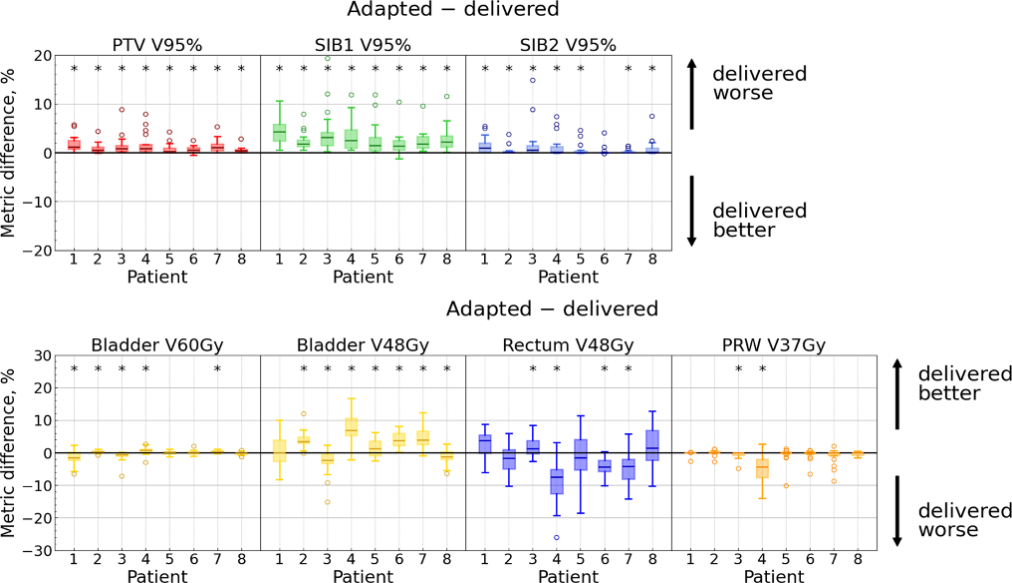
Distributions of absolute metric differences: “adapted–delivered” dose. Each subplot represents one metric, and each box corresponds to a single patient. Each box extends from the first quartile (Q1) to the third quartile (Q3), with a line indicating the median. Dots represent outliers (data points lying outside the interval $[Q1-1.5IQR, Q3+1.5IQR]$, where IQR denotes the interquartile range). Significant differences ($p < 0.05$) are marked with an asterisk

Statistically significant differences between adapted and delivered dose distributions were observed for most patients in the *target volume* metrics: PTV/SIB1/SIB2 V95% and SIB1 D95% yielded better results with the adapted plan on CBCT1 (see Fig. 5 as well as Fig. 4 in the Supplement). In contrast, no significant differences were noted for PTV/SIB2 D95% in most cases, with few exceptions. When significant differences were observed for PTV D95% and SIB2 D95%, adp@cbct1 demonstrated better results. The only instance where a target volume metric was significantly better with adp@cbct2 occurred for SIB2 D95% for patient 6; however, the median difference for this case was only 0.25% (here and below, we refer to absolute changes, e.g., percentage points).

**Fig. 6:**
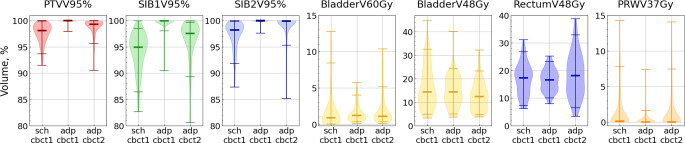
Metric distributions for all 155 fractions. Medians are shown with thick lines. For each violin plot, maximum and minimum values as well as 5% and 95% quantiles are shown

Considering the entire cohort, all target volume metrics significantly degraded with the delivered dose compared to the adapted one, as evidenced by positive medians of the difference distributions (Table 2 and Fig. 6). However, PTV and SIB2 metrics stayed within the alternative constraints for most sessions with the delivered dose.

**Table 2:** Percentage of sessions with an unmet *alternative* constraint for a specific metric for each dose distribution: non-adapted, adapted, and delivered as well as median and range of metric difference distribution with the corresponding p-values (obtained from the Wilcoxon signed-rank test for paired data with a two-sided alternative hypothesis) for each pair of dose distributions (adapted–delivered and delivered–non-adapted) across the entire cohort

Metric		Sessions with unmet constraint, %			Metric differences, percentage points			
					Adapted–delivered		Delivered–non-adapted	
		Non‑adapted	Adapted	Delivered	Median [range]	*p*-value	Median [range]	*p*-value
PTV	V95%	10	0.0	3.9	0.63 [−0.5 … 8.9]	$<0.001$	0.98 [−8.3 … 8.2]	$<0.001$
	D95%	10	0.0	3.9	1.05 [−3.7 … 23.7]	$<0.001$	3.95 [−21.3 … 25.6]	$<0.001$
SIB1	V95%	50	1.9	21	2.11 [−1.2 … 19.4]	$<0.001$	2.46 [−14.9 … 15.8]	$<0.001$
	D95%	50	1.9	21	1.15 [−0.8 … 21.6]	$<0.001$	2.20 [−28.6 … 30.0]	$<0.001$
SIB2	V95%	19	0.0	4.5	0.09 [−0.19 … 14.8]	$<0.001$	1.31 [−12.0 … 12.6]	$<0.001$
	D95%	19	0.0	4.5	0.017 [−1.1 … 9.4]	0.002	1.33 [−9.7 … 12.1]	$<0.001$
Bladder	V60Gy	9.7	1.3	5.2	−0.026 [−7.1 … 2.7]	0.712	0.35 [−11.9 … 8.8]	0.052
	V48Gy	20	3.9	3.9	2.36 [−15.2 … 16.6]	$<0.001$	−3.15 [−29.0 … 16.5]	$<0.001$
	V40Gy	1.3	0.0	0.0	3.25 [−16.9 … 19.6]	$<0.001$	−4.13 [−36.2 … 18.8]	$<0.001$
Rectum	V48Gy	0.0	0.0	3.2	−1.60 [−26.1 … 12.7]	0.002	1.05 [−19.3 … 28.3]	0.009
	V24Gy	0.0	0.0	0.6	−1.55 [−30.0 … 14.1]	0.002	−1.87 [−22.5 … 30.3]	$<0.001$
PRW	V37Gy	10	0.7	11	0.0 [−14.1 … 2.7]	0.003	0.0 [−10.0 … 12.6]	0.796
	V30Gy^a^	30	22	41	−0.71 [−15.7 … 6.4]	$<0.001$	−0.12 [−22.6 … 14.5]	0.763

^a^PRW V30Gy is not clinically relevant and has low priority during plan calculation

There are two main factors affecting the* bladder* metrics: (1) the adapted plan is calculated based on CBCT1 (meaning that adp@cbct1 is expected to be better than adp@cbct2), but (2) the bladder continues to fill between CBCT1 and CBCT2 (which means that adp@cbct2 is expected to be better than adp@cbct1). For some patients, one factor predominated, while for others, the other factor played a more significant role. This may explain the opposing results observed in some patients (Fig. 5). If the bladder did not fill much between the two CBCT scans, bladder metrics could be worse with adp@cbct2. Patient 8 could serve as example here: he exhibited a very small bladder volume increase (19.5 ml on average), and bladder V48Gy degraded with adp@cbct2, while bladder V60Gy and V40Gy changed nonsignificantly. Considering the entire cohort, only the V48Gy and V40Gy metrics exhibited significant improvement with the delivered dose (Table 2).

The *rectum* may also change its shape between CBCT1 and CBCT2, but no consistent trend is expected regarding whether the rectum volume increases or decreases. Consequently, no definitive behavior of rectum metrics can be predicted based solely on rectum volume. However, as the adapted plan is based on CBCT1, it is expected that adp@cbct1 would perform better or, at least, not worse. This was confirmed for 7/8 patients (Fig. 5). For the entire cohort, both V48Gy and V24Gy were significantly worse with adp@cbct2 (Table 2), but they still met objectives for most sessions.

As mentioned above, V37Gy for the *PRW *is often zero. Therefore, we focused on V30Gy, using V37Gy as a supplementary measure. For four patients, V30Gy exhibited a statistically significant difference, showing for one patient a small improvement with adp@cbct2, while for 3 others, a degradation (Fig. 4 in the Supplement). For the entire cohort, V30Gy was significantly better with adp@cbct1 (Table 2). V37Gy yielded a significant difference only in two patients. Frequent zero values of V37Gy led to a counterintuitive result: for the entire cohort, the Wilcoxon test revealed a significant difference between these two dose distributions ($p = 0.003$), but the median difference was exactly 0. The mean difference was −1.0% though, which means that adp@cbct1 outperformed adp@cbct2.

#### Delivered (adaptive) vs. non-adapted dose distribution

This comparison addresses the clinical question of whether the adaptation remains beneficial in the face of the intra-adaptational anatomical changes.

It is important to note that the cohort is relatively small (8 patients), and we cannot cover all possible relationships between organ volumes on pCT and CBCTs evenly. These relationships strongly influence results of the “non-adapted vs. delivered” comparison (discussed below). Among the cohort, 3/8 patients had $V_\mathrm{pCT,\,bladder} < \mathrm{Median}(V_\mathrm{CBCT1,\,bladder})$, also 3/8 patients had $V_\mathrm{pCT,\,prostate} < \mathrm{Median}(V_\mathrm{CBCT1,\,prostate})$, and only 1 patient had both the prostate and the bladder volumes smaller on the pCT, while 3 patients had both volumes bigger on the pCTs.

Two sets of anatomical changes play a role in this comparison. First, the changes between pCT and CBCT1 affect the scheduled plan on CBCT1, worsening it compared to the same plan on the pCT (which almost certainly meets all the objectives). On the other hand, the changes occurring between CBCT1 and CBCT2 could worsen adp@cbct2 compared to adp@cbct1. The key comparison is the following: which of these two sets of changes has a stronger influence? Intuitively, the changes between CBCT1 and CBCT2 should be smaller than those between pCT and CBCT1. Therefore, we expect adp@cbct2 to be better/not worse than sch@cbct1.

**Fig. 7:**
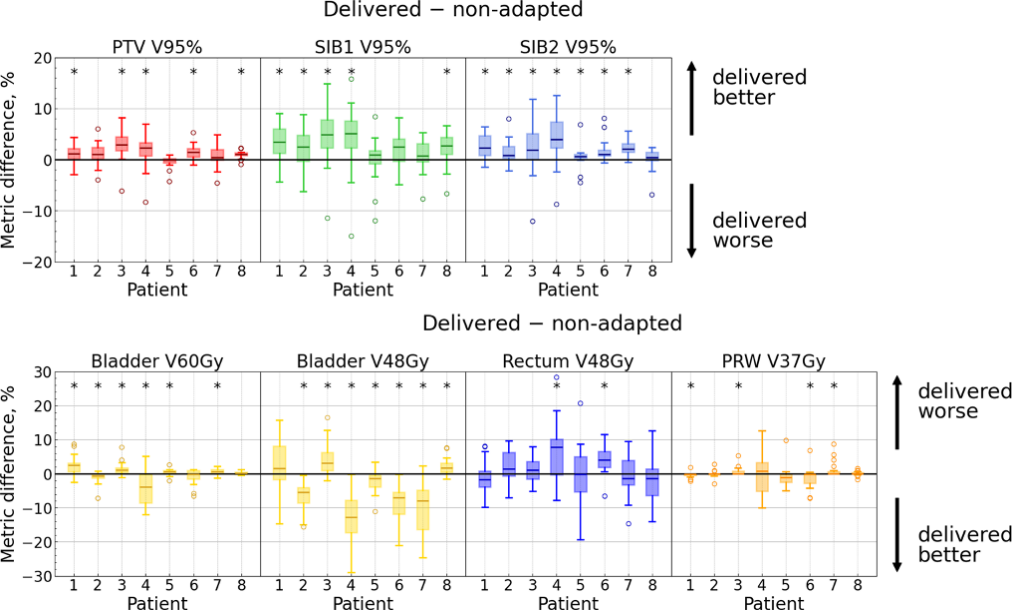
Distributions of absolute metric differences: “delivered–non-adapted” dose. Each subplot represents one metric, and each box corresponds to a single patient. Each box extends from the first quartile (Q1) to the third quartile (Q3), with a line indicating the median. Dots represent outliers (data points lying outside the interval $[Q1-1.5IQR, Q3+1.5IQR]$, where IQR denotes the interquartile range). Significant differences ($p < 0.05$) are marked with an asterisk

This expectation was quantitatively proven for all the *target volume* metrics for each patient individually (see Fig. 7 as well as Fig. 5 in the Supplement). Furthermore, when considering the entire cohort, all target volume metrics significantly increased by 1–4% with the delivered dose distribution (Table 2 and Fig. 6).

Considering individual patients, the *OAR* metrics showed inconsistent results. Only for the middle-dose bladder metrics did the majority of patients exhibit improvement with the delivered dose (see Fig. 7 as well as Fig. 5 in the Supplement). Moreover, for the entire cohort, bladder V48Gy and V40Gy as well as rectum V24Gy were lower with adp@cbct2 (Table 2). At the same time, bladder V60Gy and both PRW metrics yielded no statistically significant differences. Rectum V48Gy, however, was better with the non-adapted dose.

### Unmet constraints

As discussed above, the adapted dose is expected to yield better outcomes than the delivered dose. Although this was confirmed for most metrics, a key question is how often is this difference large enough to result in an unmet objective with the delivered dose? Table 2 presents the percentages of sessions with unmet constraint for all three dose distributions. We observed an increase in constraint failures between the adapted and the delivered dose for all metrics (except for bladder V48Gy and V40Gy). The increase varied from 0.6 to 19% (percentage points) among the different metrics. A high increase of 10–19% was observed for the PRW and SIB1 metrics, while for all other metrics, the increase did not exceed 4.5%. A high number of sessions with a PRW V30Gy constraint failure (even with the adapted dose) could be explained by a low priority of this metric during plan calculation^2^.

Additionally, we assessed the number of sessions in which a goal for a given metric (an alternative goal, when it exists) was satisfied with adp@cbct1 but not with adp@cbct2. These session counts are summarized in Table 1 in the Supplement.

There were 15 sessions in which only both SIB1 goals were not achieved with the delivered dose (being achieved with the adapted one). The possible reason for this is a steep gradient in the dose in the craniocaudal direction, which is additionally “reinforced” by the definition of the SIB1 used in CHHiP [10]: SIB1 and PTV contours are identical almost along the entire contour, except for seminal vesicle area and posteriorly. But the isodose lines for SIB1 and PTV differ everywhere: the 95% prescribed-dose isoline for PTV includes more area than the one for SIB1. In the case when the prostate contour would be slightly shifted downwards on CBCT2 with respect to CBCT1 (as depicted in Fig. 3 in the Supplement), the probability of the SIB1 goals being unmet is the highest among all target volumes.

## Discussion

The target volume contours were derived from the prostate and the seminal vesicle contours. Due to the short time between CBCT1 and CBCT2, any changes in the volumes of these organs are more likely attributable to contouring quality rather than genuine anatomical changes. The prostate contouring demonstrated a high degree of consistency between the two scans (the mean volume difference per patient ranged from 0.01 to 0.73 ml). However, the observed differences for the seminal vesicles exceeded those for the prostate: the mean difference ranged from 0.12 to 2.8 ml across individual patients. This discrepancy likely stemmed from the contouring process: while high consistency was achieved for the prostate, due to rigid fusion of the CBCT1 contour with CBCT2 focusing specifically on the prostate, the seminal vesicles received comparatively less focus and were recontoured on CBCT2 to a higher extent than the prostate. While the prostate contour is crucial, as it forms a foundation for the calculated target volumes (TVs), the impact of the seminal vesicles is relatively minor, as only up to 2 cm of their base is included in the TV contours. Consequently, the observed mean differences in seminal vesicle volumes between CBCT1 and CBCT2 presumably had a minimal impact on overall plan quality.

The *target volume* metrics are the primary focus during plan calculation/optimization. As expected, all these metrics were better/not worse with adp@cbct1 than with adp@cbct2 for individual patients, as there were no anatomical changes anticipated, which could improve the metrics. While the medians of the metric changes were small for the entire cohort of patients (Table 2), they were statistically significant. A decrease in target dose between the planned and delivered dose distributions has been reported by other authors [21–24].

Therefore, the important question is whether these metrics still meet the objectives with adp@cbct2. Indeed, in 9/155 sessions, the PTV and/or SIB2 alternative objectives were unmet with the delivered dose, while being met with the adapted one (see Table 1 in the Supplement). Such a low number of unmet target goals is consistent with those reported by other authors [24, 25]. We observed that PTV or SIB2 constraints were unmet in up to two sessions per patient with the delivered dose (SIB1 constraints were unmet more often, as discussed above), which may be relevant for tumor control. However, the real issue is whether the adaptation remains beneficial, e.g., whether the delivered dose is still better than the non-adapted one. For individual patients, all target volume metrics were either better with adp@cbct2 than those with sch@cbct1 or did not show any significant difference (Fig. 7). The percentage of sessions with unmet objectives for a specific target volume metric experienced a decrease in the range of 6 to 29% (percentage points) between the non-adapted and the delivered dose distribution (Table 2).

The metrics for *OARs *demonstrated inconsistent results. It is important to note that adp@cbct1 occasionally performed worse for OARs than sch@cbct1 (see Fig. 6 in the Supplement). Naturally, there was a valid reason for selecting the adapted plan over the scheduled one, such as improved target volume metrics. However, it is unsurprising that adp@cbct2 performed worse than sch@cbct1 in those cases, given that even adp@cbct1 was already inferior for certain OAR metrics.

For the *bladder*, both the organ volume and its changes play a significant role. Although patients were instructed to maintain a full bladder during each treatment session and at the pCT, significant differences in the bladder volume were often observed (Fig. 3). As was quantitatively proven [11], a bigger bladder generally improves bladder sparing. However, several effects can arise if the bladder volume differs strongly among the pCT and both CBCTs. If a bladder volume was smaller in pCT than in CBCT1, bladder sparing may be better with sch@cbct1 than with adp@cbct1 (see Fig. 6 in the Supplement), because the scheduled plan was calculated based on a small bladder and is now applied to the CBCT1 image with a larger bladder. Whether sch@cbct1 will also outperform adp@cbct2 in this case remains difficult to predict definitively. Moreover, if adaptation is performed for a bladder larger than in pCT, the optimization algorithm may not “strive as hard” to obtain good bladder protection because of the larger volume, which makes it easier to achieve the objectives. Now, the trouble is that not only the bladder volume changes, but we have seen that the prostate volume and hence the target volumes could increase as well. This effect will artificially increase the dose to the bladder (and/or to the rectum) and, hence, counteract the improvement due to the bigger bladder (an example of such a session is given in Fig. 2 in the Supplement).

We can expect the bladder volume in CBCT2 to be larger than that in CBCT1 (which is corroborated by the data: the bladder was more filled on CBCT2 in 150/155 sessions), while the prostate volume would be the same in both CBCTs. Therefore, we would expect the bladder metrics on CBCT2 to be better than on CBCT1, if the bladder volume notably increases. We observed that bladder V48Gy and V40Gy significantly decreased for 5/8 patients, while V60Gy decreased only for 3/8 patients (Fig. 5). Considering all sessions together, bladder V48Gy and V40Gy significantly decreased on CBCT2, while V60Gy changed nonsignificantly. A similar observation was made in [7] for 6 patients (150 sessions) with cervical cancer. The authors evaluated the impact of intrafractional changes of OARs (between pre- and posttreatment MRI) on their dose. A negative intrafractional dose change for bladder observed for D50% and D_mean_ (but not for D2%) was explained by the increased bladder volume.

If there is no marked bladder filling between CBCT1 and CBCT2, adp@cbct2 may be worse, since the effects of filling (e.g., sparing improvement) and anatomical changes (e.g., sparing degradation) counteract each other, and which effect predominates depends on the patient and the exact anatomy.

For the *rectum *and *posterior rectal wall *metrics, only few patients exhibited significant differences, and no systematic improvement/deterioration could be observed in these metrics for the entire cohort. This is physiologically plausible and similar to the results published by [7], where a low mean intrafractional change in D_mean_ for rectum was reported.

We observed notable variations in the rectum shape for patients with meteorism. In fact, for all six sessions with rectum objective failures with adp@cbct2, the reason could be always attributed to a downward movement of an air pocket in the rectum between CBCT1 and CBCT2, compressing the prostate and the seminal vesicles (Fig. 2d). In addition to failed rectum objectives, this also caused unmet bladder (in one of all 11 cases with unmet bladder objectives), PTV (3/6 cases), and SIB2 (2/7 cases) objectives. In [21], the authors compared the planned and delivered dose distributions for prostate radiotherapy with an MR linac. The delivered dose accounted for motion during treatment delivery. They reported a decrease of CTV D99% by 7% and an increase of rectum V60Gy by 8.2% in the case when a gas pocket passed through the rectum. A similar observation was made in [23] for patients with prostate cancer undergoing MR-guided radiotherapy. The dosimetric impact of prostate motion was small in most cases. However, in one fraction, where a gas pocket caused a large intrafractional motion, rectum D3% and bladder D3% increased by 0.4 Gy and 0.8 Gy, respectively, although the CTV dose changed only slightly.

Thus, for all six sessions with rectum objective failures in this study, the reason was physiological and the same in all cases (meteorism occurring between CBCT1 and CBCT 2); bladder, PTV, and SIB2 objectives were rather mostly unmet due to poor fusion or image quality, and only few cases were related to physiological causes (again gas in the rectum).

### Clinical appraisal of results

Taking a step back from the results, we should first distinguish two very important and very different aspects:

First and foremost, in online adaptive radiotherapy, there are real anatomical and physiological effects, which we can for the first time observe and integrate into an adapted, reoptimized plan.

Secondly, and not to be confused with the former, there are differences in what we see, not in what there is. In particular, this arises from the image quality of the CBCTs with respect to the planning CT and—even more importantly—the use of planning MRI. If contouring of the prostate is performed as it is in our institution, the adapted contours on CBCT (regardless of whether CBCT1 or CBCT2) will be larger than the original planning CT- and MRI-based contours. This directly entails an apparent underdosage of the scheduled plan on the CBCT, which is of course spurious and arises from an overestimation of the prostate volume. Therefore, even in the complete absence of any anatomical variation between pCT and CBCT1, the scheduled plan will appear to perform worse in terms of target coverage and better regarding dose to the organs at risk. The adaptation process to some degree only counteracts this effect, bringing the apparent DVH metrics back on course (but relying on a target contours set based on a different imaging modality). Taking this thought to the end, paradoxically, adaptation would make a good plan worse. Only if the real anatomical variations are substantial enough to really warrant adaptation (even in the face of identical contouring algorithms) will the net effect be an improvement. Fact is, a number of studies [3, 4] have proven the utility of plan adaptation with improved target coverage (despite a possibly larger target) and improved OAR sparing, so the advantage evidently prevails.

Now looking at the change between CBCT1 and CBCT2, this should show real anatomical differences only, since the same imaging modality is used. Therefore, a comparison of plans on CBCT1 and CBCT2 will not be biased so much by contouring differences. However, when clinically evaluating the delivered dose, we feel we must emphasize that the so-called delivered dose displayed in the system is the adapted plan as it would result on CBCT2 anatomy but with DVH metrics evaluated for CBCT1 contours! This is obviously misleading. The difference between the real delivered dose and the delivered dose provided by the Ethos system (“reconstructed dose”) is presented in the Supplement (see Fig. 5 in the Supplement). This being said, the intention of the designers to provide a real delivered dose is laudable, since anatomical variations indeed do arise during the adaptation process, as we have seen in this study. Therefore, the only difficulty is in determining the real delivered dose and the true DVH metrics, which we have done exemplarily here (the difference between the real delivered dose and the delivered dose provided displayed in the system is presented in the Supplement).

What we can see is that while the delivered plan is actually somewhat worse than the first adaptation, it is still better than the original plan without any ART at all. Therefore, even though adaptation does take some time, and variations in anatomy can occur in this interval, adaptation is still worthwhile with regard to creating the best optimal treatment plan for the patient on each individual treatment day.

## Conclusion

We quantitatively proved that plan adaptation is beneficial for the PTV/SIB1/SIB2 metrics for each individual patient even despite the intra-adaptational anatomical changes. The bladder demonstrated a strong dependence on bladder volume changes between pCT, CBCT1, and CBCT2. Since the bladder continued to fill between CBCT1 and CBCT2, it was most likely that the bladder metrics improved with the delivered dose in comparison to the adapted one. Moreover, the adaptation remained beneficial for the bladder even with intra-adaptational anatomical changes. The rectum and PRW metrics were patient dependent and showed no clear trend.

The most common reasons for an objective failure with the delivered dose were not anatomical and included, for example, an imperfect fusion between CBCT1 and CBCT2. Unmet rectum metric objectives (6/155 sessions) always resulted from a changed rectum shape (meteorism happening between CBCT1 and CBCT2). The same issue sometimes led to unmet bladder and/or target volume objectives. Thus, intra-adaptational anatomical changes worsened the performance of the adapted plan on CBCT2, and they were partially responsible for unmet objectives.

Despite these changes, the clinically delivered adapted plan was still better than the scheduled plan, which proves the benefits of online ART even in the realistic clinical setting.

## Supplementary Information

Supplementary materials

## Footnotes

1 The algorithm includes the following steps: (1) the rectum contour is expanded by 0.2 cm in the anterior–posterior direction and laterally by 5 cm. The aim of this is to even out the edges and create a smooth, linear anterior border to the PRW; (2) The resulting help structure is then shrunk by 1.3 cm in the posterior direction, thus eliminating the posterior 1.1 cm of the rectum. The freely selected parameter of 1.3 cm is an empirical value that is appropriate for all regarded clinical cases. (3) The original rectum is then cropped by this second structure, so that only parts contained within the rectum (posterior part) are retained.

2 The priority scale expands from 1 to 4, whereby all considered metrics have the priority level 1 or 2, while PRW V30Gy has level 3.
