# Supplementary material for: Intra-adaptational changes in online adaptive radiotherapy: from the ideal to the real dose
Source: Strahlenther Onkol. 2025 Jul 15;201(11):1170–84. doi: 10.1007/s00066-025-02425-9 (PMC12546291; doi:10.1007/s00066-025-02425-9)
Supplement: Supplementary file 1 — Supplementary materials [file 66_2025_2425_MOESM1_ESM.pdf]

Intra-adaptational changes in online adaptive radiotherapy: from the ideal to the real dose

1 Supplementary Materials

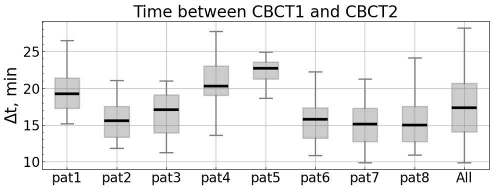

Supplementary Figure 1 Time difference between CBCT1 and CBCT2 for each patient individually.

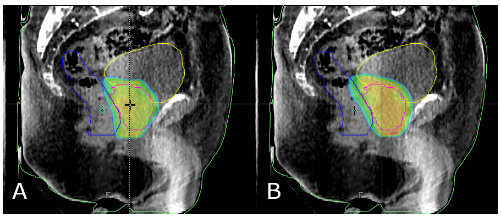

Supplementary Figure 2 Dose distributions for a non-adapted and an adapted plan on the same CBCT1 image. A: the non-adapted dose distribution, B: the adapted one. The contours are CBCT1 contours: the prostate in magenta, the bladder in yellow, and the rectum in blue. Patient 3, session 2.

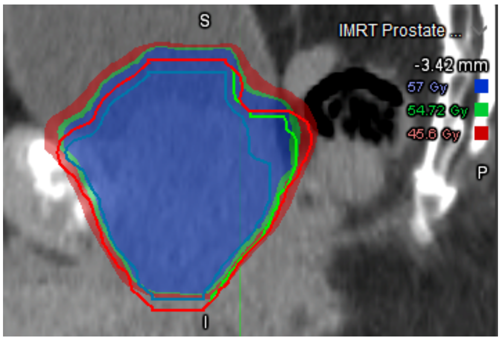

Supplementary Figure 3 Example of unmet goals for SIB1 with adp,cbct2 while being met for adp,cbct1 (all other contraints are satisfied on both CBCTs). CBCT2 contours are depicted on the pCT image, the isodose lines correspond to the adapted plan. PTV contour is red, SIB1 – light green, SIB2 – light blue. The isodose areas are filled with corresponding colors.

Supplementary Table 1 Number of sessions in which goals were satisfied/not satisfied for each dose. For example, the column titled "+ + –" contains the number of sessions, in which a specific metric goal was met with both non-adapted and adapted, but not – with delivered dose distribution. In the last three columns, the percentage of sessions with non-met specific objective are listed.

Dose:	Is the goal satisfied?							
non-adapted	+	+	+	+	–	–	–	–
adapted	+	+	–	–	+	+	–	–
delivered	+	–	+	–	+	–	+	–
Metric	Number of sessions							
PTV V95%	134	5	0	0	15	1	0	0
D95%	134	5	0	0	15	1	0	0
SIB1 V95%	63	12	1	1	59	18	0	1
D95%	63	12	1	1	59	18	0	1
SIB2 V95%	121	5	0	0	27	2	0	0
D95%	121	5	0	0	27	2	0	0
Bladder V60Gy	130	8	2	0	15	0	0	0
V48Gy	119	5	0	0	25	0	5	1
V40Gy	153	0	0	0	2	0	0	0
Rectum V48Gy	150	5	0	0	0	0	0	0
V24Gy	154	1	0	0	0	0	0	0
PRW V37Gy	111	10	0	0	9	4	0	1
V30Gy	65	20	0	9	9	11	5	16

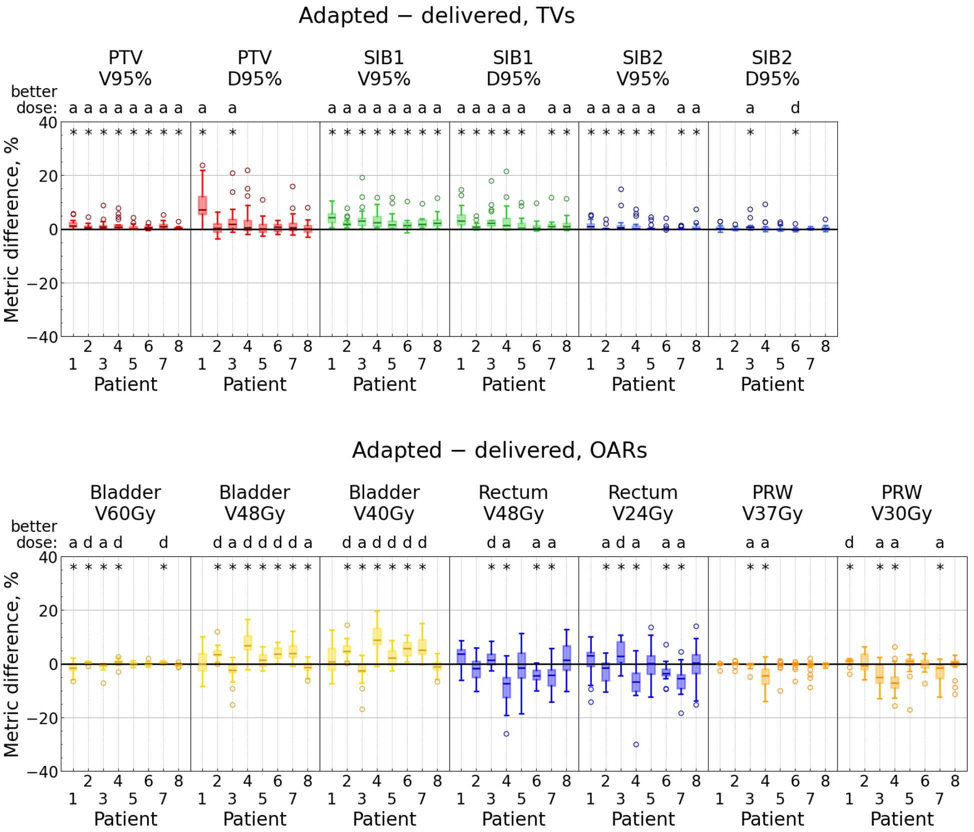

Supplementary Figure 4 Distributions of absolute metric differences: "adapted - delivered" dose. Each subplot represents one metric, and each box corresponds to a single patient. Each box extends from the first quartile (Q1) to the third quartile (Q3), with a line indicating the median. Dots represent outliers – data points lying outside the interval $[Q1 - 1.5IQR, Q3 + 1.5IQR]$, where IQR denotes the interquartile range). Significant differences ($p < 0.05$) are marked with *. Better dose distribution for each metric and each patient is labeled with corresponding letter on top of the plot: "a" for adapted dose and "d" for delivered dose.

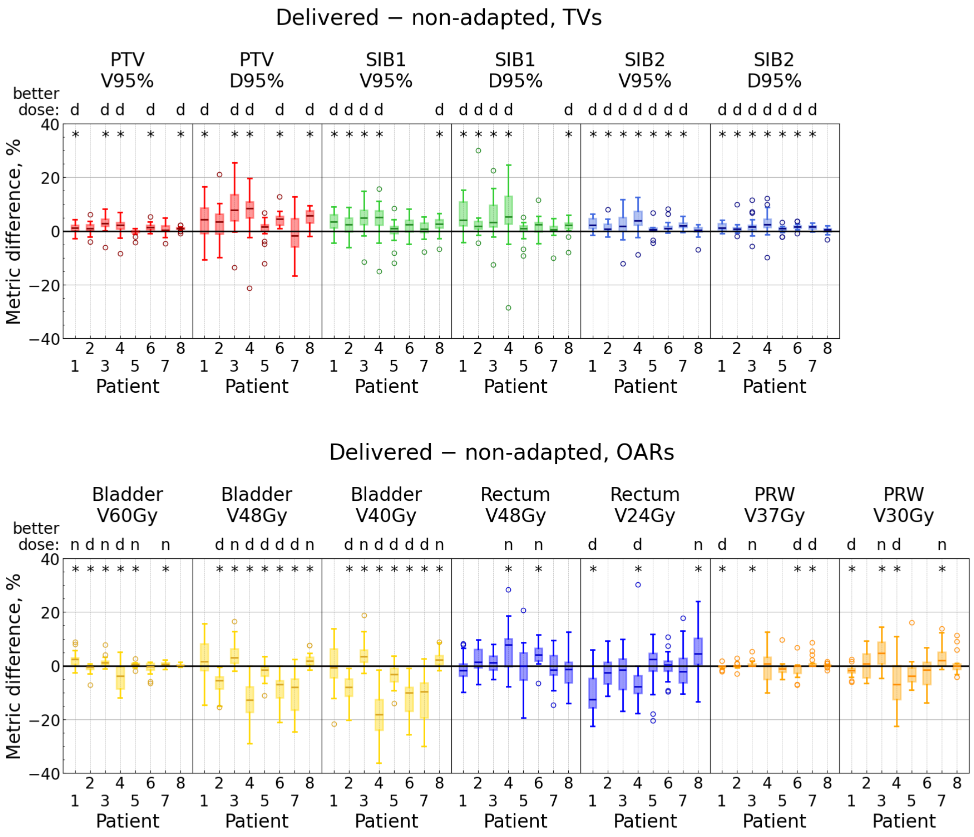

Supplementary Figure 5 Distributions of absolute metric differences: "delivered - non-adapted" dose. Each subplot represents one metric, and each box corresponds to a single patient. Each box extends from the first quartile (Q1) to the third quartile (Q3), with a line indicating the median. Dots represent outliers – data points lying outside the interval $[Q1 - 1.5IQR, Q3 + 1.5IQR]$, where IQR denotes the interquartile range). Significant differences ($p < 0.05$) are marked with *. Better dose distribution for each metric and each patient is labeled with corresponding letter on top of the plot: "d" for delivered dose and "n" for non-adapted dose.

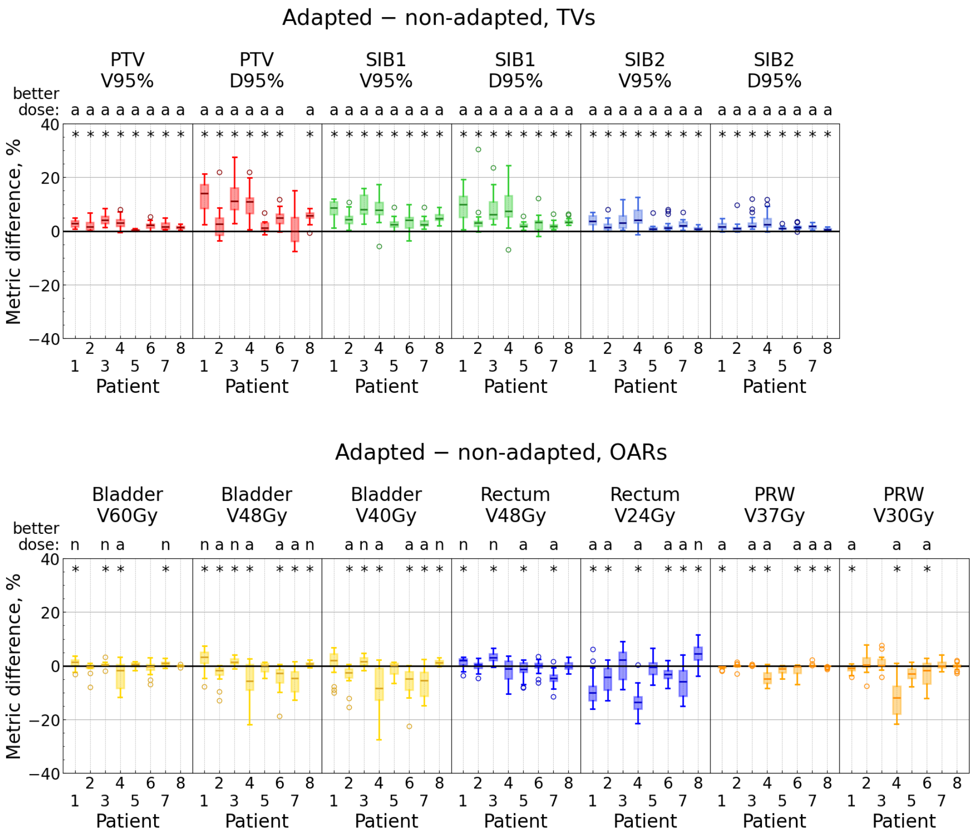

Supplementary Figure 6 Distributions of metric differences: ”adapted - non-adapted” dose. Each subplot represents one metric, and each box corresponds to a single patient. Each box extends from the first quartile (Q1) to the third quartile (Q3), with a line indicating the median. Dots represent outliers – data points lying outside the interval $[Q1 - 1.5IQR, Q3 + 1.5IQR]$, where IQR denotes the interquartile range). Significant differences ($p < 0.05$) are marked with *. Better dose distribution for each metric and each patient is labeled with corresponding letter on top of the plot: ”a” for adapted dose and ”n” for non-adapted dose.

1.1 Delivered dose vs reconstructed dose provided by Ethos

The Ethos treatment system provides so-called delivered or reconstructed dose. According to the "Ethos Admin and Physics" documentation, the following information is used to calculate DVHs for the reconstructed dose:

1. The system registers the planning CT (pCT) with the last taken CBCT and deforms the pCT using the Velocity image deformation algorithm to create a synthetic CT (sCT), in which each CBCT voxel's Hounsfield Unit (HU) is replaced with the corresponding HU from the pCT.
2. The (adapted) plan, the delivered monitor units, and the isocenter position are extracted from the treatment records.
3. A rigid registration is performed between the CBCT1 and the CBCT2, such that the contours defined on the CBCT1 are used for the DVH calculation.

As a result, any intra-adaptational anatomical changes are reflected only through HU changes, not through updated contours. This leads to the discrepancies between the actual delivered dose and the reconstructed dose displayed by the system. These discrepancies are illustrated in Supplementary Figure 7.

In the most cases, the treatment system significantly overestimates the dose to the targets, misleadingly indicating better coverage. It also overestimates the dose to the bladder, while in reality the bladder sparing is better. For the rectum and the posterior rectal wall, the differences between reconstructed and delivered doses are statistically significant in fewer cases.

The comparison between reconstructed and delivered doses closely mirrors the comparison between adapted and delivered doses: the reconstructed dose displayed by the system closely resembles the adapted dose. This is expected, as only the HU changes in CBCT2 are considered, while changes in organ contours could not be taken into account.

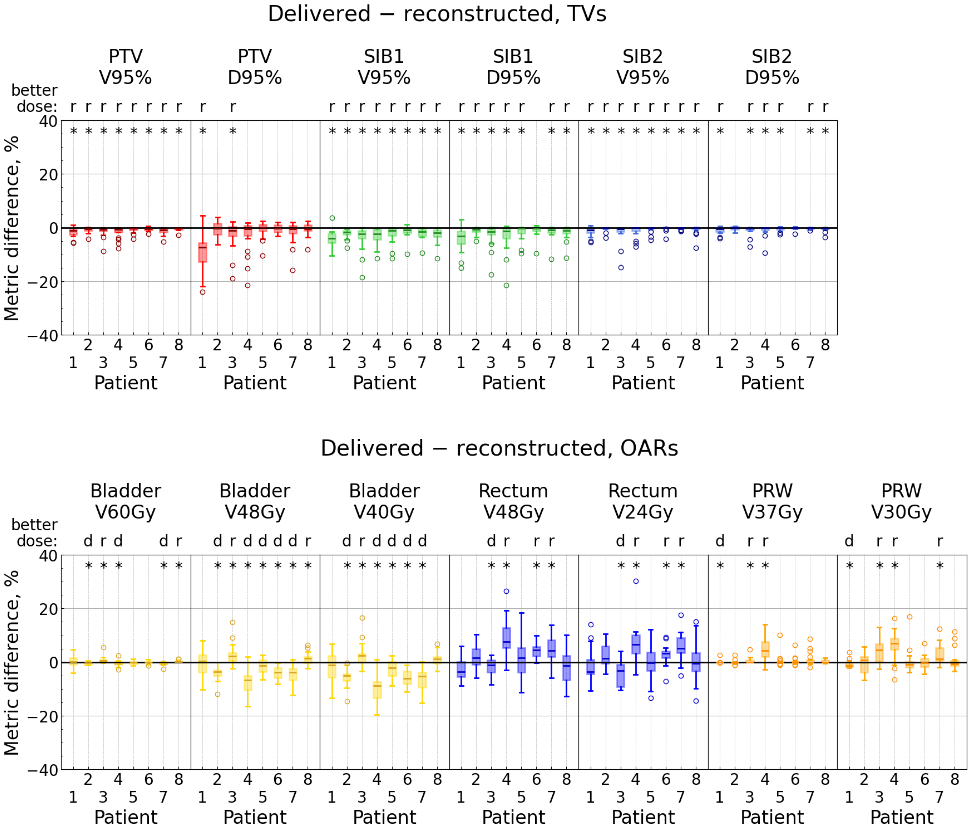

Supplementary Figure 7 Distributions of absolute metric differences: "delivered - reconstructed" dose (where the reconstructed dose is the delivered dose displayed in the treatment system, also referred as "reconstructed dose"). Each subplot represents one metric, and each box corresponds to a single patient. Each box extends from the first quartile (Q1) to the third quartile (Q3), with a line indicating the median. Dots represent outliers – data points lying outside the interval $[Q1 - 1.5IQR, Q3 + 1.5IQR]$, where IQR denotes the interquartile range). Significant differences ($p < 0.05$) are marked with *. Better dose distribution for each metric and each patient is labeled with corresponding letter on top of the plot: "d" for delivered dose and "r" for reconstructed dose.
